# Growth hormone modulates hypothalamic inflammation in long‐lived pituitary dwarf mice

**DOI:** 10.1111/acel.12382

**Published:** 2015-08-12

**Authors:** Marianna Sadagurski, Taylor Landeryou, Gillian Cady, John J. Kopchick, Edward O. List, Darlene E. Berryman, Andrzej Bartke, Richard A. Miller

**Affiliations:** ^1^ Department of Internal MedicineDivision of Geriatric and Palliative MedicineUniversity of MichiganAnn ArborMIUSA; ^2^ Department of Pathology and Geriatrics CenterUniversity of MichiganAnn ArborMIUSA; ^3^ Edison Biotechnology InstituteOhio UniversityAthensOHUSA; ^4^ Department of Internal Medicine–Geriatrics ResearchSouthern Illinois University School of MedicineSpringfieldILUSA

**Keywords:** aging, dwarf mice, growth hormone, hypothalamus, inflammation, longevity

## Abstract

Mice in which the genes for growth hormone (GH) or GH receptor (GHR ^−/−^) are disrupted from conception are dwarfs, possess low levels of IGF‐1 and insulin, have low rates of cancer and diabetes, and are extremely long‐lived. Median longevity is also increased in mice with deletion of hypothalamic GH‐releasing hormone (GHRH), which leads to isolated GH deficiency. The remarkable extension of longevity in hypopituitary Ames dwarf mice can be reversed by a 6‐week course of GH injections started at the age of 2 weeks. Here, we demonstrate that mutations that interfere with GH production or response, in the Snell dwarf, Ames dwarf, or GHR ^−/−^ mice lead to reduced formation of both orexigenic agouti‐related peptide (AgRP) and anorexigenic proopiomelanocortin (POMC) projections to the main hypothalamic projection areas: the arcuate nucleus (ARH), paraventricular nucleus (PVH), and dorsomedial nucleus (DMH). These mutations also reduce hypothalamic inflammation in 18‐month‐old mice. GH injections, between 2 and 8 weeks of age, reversed both effects in Ames dwarf mice. Disruption of GHR specifically in liver (LiGHRKO), a mutation that reduces circulating IGF‐1 but does not lead to lifespan extension, had no effect on hypothalamic projections or inflammation, suggesting an effect of GH, rather than peripheral IGF‐1, on hypothalamic development. Hypothalamic leptin signaling, as monitored by induction of pStat3, is not impaired by GHR deficiency. Together, these results suggest that early‐life disruption of GH signaling produces long‐term hypothalamic changes that may contribute to the longevity of GH‐deficient and GH‐resistant mice.

**Abbreviations**
CR: calorie restriction
GH: growth hormone
GHR: growth hormone receptor
CNS: central nervous system
LepR‐b: leptin receptor
Stat3: signal transducer and activator of transcription 3
POMC: proopiomelanocortin
ARH: arcuate nucleus of the hypothalamus

Among genetically altered long‐lived mice, animals with growth hormone (GH) deficiency, such as hypopituitary Ames (df/df) and Snell (dw/dw) dwarf mice, or GH insensitivity, such as the GH receptor (GHR) gene‐disrupted mouse (GHR^−/−^), stand out by virtue of the magnitude of life extension and consistency of the findings across sexes, genetic background, and diets (Bartke & Brown‐Borg, 2004; Bartke, 2008). These remarkably long‐lived animals are characterized by reduced postnatal growth rate, delayed development, and reduced adult body size (Junnila *et al*., 2013). Mice lacking Ghrh, or Ghrhr (Flurkey *et al*., 2001; Sun *et al*., 2013), are also long‐lived, as are mice lacking PAPP‐A, a protease specific for IGF‐1‐binding proteins (Conover & Bale, 2007), further implicating GH and/or IGF‐1 tonicity in the regulation of mammalian aging rate and lifespan.

Growth hormone is active in the central nervous system, influencing feeding behavior and a sense of well‐being in humans (Blissett *et al*., 2000). GH feeds back on the hypothalamus and regulates its own secretion (Kamegai *et al*., 1996). The arcuate nucleus of the hypothalamus (ARH) contains neurons that produce the anorexigenic peptide melanocyte‐stimulating hormone (MSH) and other neurons that coexpress the orexigenic peptides neuropeptide Y (NPY) and agouti‐related protein (AgRP), which regulate food intake and energy expenditure (Myers, 2004). The ARH neurons interact with pituitary cells that produce GH, through the opposing actions of somatostatin (SS) and GH‐releasing hormone (GHRH), derived from the periventricular nucleus and ARH of the hypothalamus (Muller *et al*., 1999). Systemic administration of GH induces expression of the c‐fos gene, a marker of neuronal activity, in the hypothalamic NPY and somatostatin neurons (Kamegai *et al*., 1994). The majority of NPY mRNA‐containing cells in the hypothalamic arcuate nucleus express the GHR gene, and NPY mRNA expression is downregulated in GHR^−/−^ mice (Peng *et al*., 2001), suggesting that NPY neurons in the ARH mediate the feedback effect of GH on the hypothalamus.

There is growing evidence that the first few weeks of life may be a period in which GH action can influence the course of aging and that the time window for this developmental programming of aging may be relatively short (Bartke *et al*., 2013). Ames dwarf mice injected with GH between 2 and 8 weeks of age do not live longer than littermate control mice (Panici *et al*., 2010), suggesting that the extended lifespan of Ames dwarf mice depends upon lower availability of GH during the first few weeks of life, rather than on the level of GH action after the 8th week of life. Skin‐derived fibroblasts from GH‐injected dwarf mice also failed to show the enhanced resistance to lethal stresses observed in cells from uninjected dwarf mice (Panici *et al*., 2010). In contrast, injections of GH starting at 4 weeks of age did not reduce extended lifespan in Snell dwarf (Vergara *et al*., 2004) or in Ames dwarf mice (A. Bartke, unpublished data), suggesting that the susceptibility to GH effects on longevity may be diminished shortly after weaning. Conversely, diminution of nutrient availability and IGF‐1 levels in the first few weeks of life can lead to increased lifespan. Mice derived from cross‐fostered crowded litters (CL), in which 12 pups are present (‘CL’ mice), are longer lived than mice from cross‐fostered litters of 8 pups, showing that reduced nutrient availability in the first 3 weeks of life can itself lead to extended lifespan (Sun *et al*., 2009). Crowded litter mice have lower levels of IGF‐1 at weaning than controls (Sun *et al*., 2009), consistent with the idea that low levels of GH and/or IGF‐1 early in life may have long‐lasting benefits for health and longevity. These findings indicate that some important mechanisms of aging are programmed by endocrine signaling during the first few weeks of development.

Recent evidence has suggested that hypothalamic inflammatory pathways may influence mouse lifespan in either direction (Zhang *et al*., 2013). Activation of NF‐kB dependent cytokine production and glial activity increases with age in the hypothalamus, and augmentation of this effect, either genetically or by viral‐mediated gene delivery, can shorten mouse lifespan (Zhang *et al*., 2013). More impressively, blunting hypothalamic inflammatory signals can increase lifespan in mice (Zhang *et al*., 2013). Glial activity and production of mRNA for the inflammatory cytokine TNF‐α are diminished in CL mice (Sadagurski *et al*., 2015), consistent with the idea that lower hypothalamic inflammation may modulate aging processes and lifespan in mice.

Our previous work has shown that hypothalami of CL mice, in addition to diminished steady‐state inflammation, also differ from controls in two other ways: increased density of both orexigenic and anorexigenic neural projections and increased sensitivity to leptin (Sadagurski *et al*., 2015). We therefore assessed these endpoints in three varieties of long‐lived mice with diminished GH and IGF‐1 activity. In addition, we evaluated the effects of early‐life GH injection into pituitary dwarf mice and examined hypothalami in mice in which liver‐specific disruption of GHR leads to lower IGF‐1 levels but not to lifespan extension.

## Results

### Development of ARH projections requires GH signaling

We evaluated three aspects of hypothalamic function in long‐lived mice with GH and/or IGF‐1 defects: (i) hypothalamic axonal projections; (ii) indices of inflammatory signals, each of which we had found to be altered in long‐lived CL mice; and (iii) responses to leptin injection.

**Figure 1:**
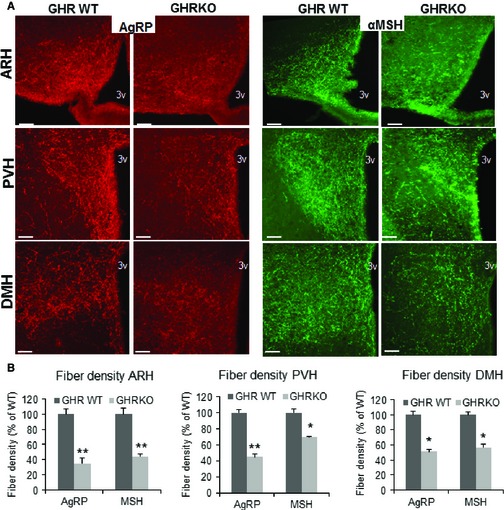
Effects of GH deficiency on hypothalamic neurocircuits. (A) Images and (B) quantification of agouti‐related peptide (AgRP) and α‐melanocyte‐stimulating hormone (α‐MSH)‐immunoreactive fibers innervating the ARH, PVH, and DMH at 6 months of age of GHR WT and GHR ^−/−^ male mice (*n* = 6/group). 3V, third ventricle. Scale bar: 200 μm. Error bars indicate SEM. *P < 0.05, **P < 0.001 versus GHR WT.

To determine whether GH‐dependent pathway leads to alterations in hypothalamic axonal projections, we analyzed the immunoreactivity of AgRP‐ and α‐MSH‐containing fibers in three main hypothalamic projection areas: the ARH, PVH (paraventricular nucleus of the hypothalamus), and DMH (dorsomedial nucleus of the hypothalamus). Because in adult animals neurons that express AgRP are restricted to NPY‐containing neurons of the ARH, AgRP‐immunoreactive (IR) fibers serve as a marker for projections from ARH NPY neurons (Bouret *et al*., 2008). The density of AgRP‐IR fibers was severely reduced in the PVH, DMH, and the ARH of 5‐month‐old GHR^−/−^ mice as compared to controls (Fig. 1A). Similar reduction in immunoreactive fibers was detected in young adult Snell dwarf mice (data not shown), as well as in Ames dwarf mice (presented below). The density of α‐MSH‐IR fibers in the PVH and DMH in GHR^−/−^ mice was also lower than in control mice (*P* < 0.03) (Fig. 1B). The distribution pattern of AgRP‐IR fibers and α‐MSH‐IR fibers in the PVH was similar among Snell dwarf, GHR^−/−^, and their respective control mice, suggesting that GH alters the density but not the pattern of innervation. It is noteworthy that this decline in neuronal projections contrasts sharply with observations in CL mice, in which the same assays showed an increase in both AgRP and α‐MSH projections (Sadagurski *et al*., 2015).

We also evaluated the effect of GH signaling on mRNA expression of hypothalamic neuropeptides critically involved in energy and glucose balance. The mRNA levels of orexigenic *NPY, AgRP*, and anorexigenic *POMC* were significantly reduced in 6‐month‐old female Snell dwarf (dw/dw) mice as compared to normal mice measured under fasting conditions (*P* < 0.0001) (Table 1). Similar results were seen in male mice (not shown). In addition, mRNA levels of these orexigenic and anorexigenic peptides were lower in 6‐month‐old female mice lacking GHR (GHR^−/−^) (*P* < 0.05) (Table 1), indicating that the change seen in the Snell dwarf mice reflected GH deficiency rather than the absence of TSH and prolactin which also characterize Snell and Ames dwarf mice.

**Table 1:** Quantitative real‐time PCR analysis of hypothalamic agouti‐related peptide (*AgRP*), neuropeptide Y (*NPY*), and proopiomelanocortin (*POMC*) mRNA expression of 6‐month‐old Snell or GHR^−/−^ female mice tested after 18 h of fasting (*n* = 8/group). Ratios are shown as mutant/control, with values < 0 indicating mRNAs expressed at lower levels in the mutants

DW/WT	DW effect (gene expression)		
	*NPY*	*POMC*	*AgRP*
Snell dwarf	0.5^a^	0.17^b^	0.13^b^
GHRKO	0.11^a^	0.1^a^	0.16^a^

a *P* < 0.05.

b *P* < 0.0001.

### GH injections reverse the disruption in ARH projections in Ames dwarf mice

**Figure 2:**
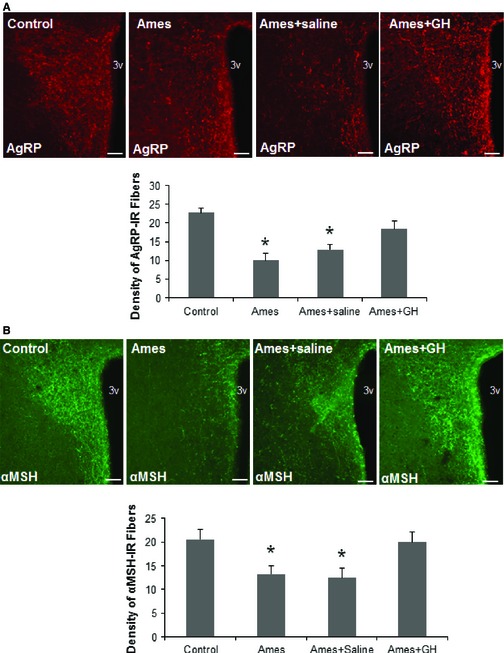
Effect of early‐life GH treatment on hypothalamic projections in Ames mice. Images and quantification of (A) AgRP and (B) α‐MSH‐immunoreactive fibers innervating the PVH at 18 months of age in control mice, Ames mice, Ames mice treated with saline injections at 2 weeks of age, and Ames mice treated with GH (*n* = 4 to 6 mice/group; all males.). 3V, third ventricle. Scale bar: 200 μm. Error bars indicate SEM. *P < 0.05 versus control.

To determine whether transient exposure to GH, early in life, can reverse or prevent the disruption in ARH projections observed in GH‐deficient mice, we evaluated 18‐month‐old Ames dwarf mice that had been subjected to a series of GH injections between 2 and 8 weeks of age, using the method previously shown to reverse or prevent lifespan extension and to augment cellular stress resistance in Ames mice (Panici *et al*., 2010). In Ames dwarf mice that had received only control (i.e., saline) injections, we found diminished levels of both AgRP and α‐MSH neurons in the PVH, consistent with the deficit noted above for Snell dwarf and GHR^−/−^ mice tested at younger ages (Fig. 2). Thus, hypothalamic re‐organization is produced on at least three background stocks (Ames, Snell, and GHR^−/−^) and is maintained at ages from 5 to 18 months. Ames dwarf mice that had been treated with GH in early life, however, did not differ from nonmutant control mice in AgRP and α‐MSH neurons in PVH (Fig. 2). Thus, as little as 6 weeks of exposure to systemic GH, early in postnatal life, leads to permanent changes in the density of hypothalamic neurons, paralleling the effects of GH injection on lifespan and cellular stress resistance (Panici *et al*., 2010) in Ames dwarf mice.

### Effect of GH on hypothalamic inflammation during aging

**Figure 3:**
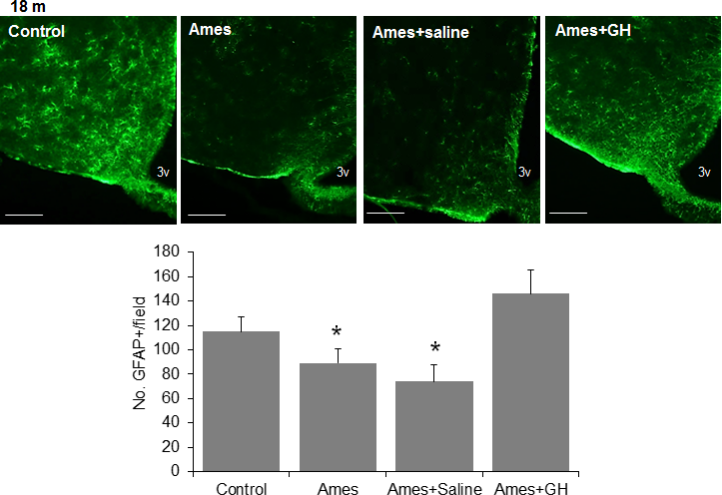
Hypothalamic astrogliosis in GH‐treated Ames mice. Representative images of astrocytes identified by immunofluorescent detection of GFAP protein in coronal sections of hypothalamus obtained from 18‐month‐old male control, Ames, and Ames treated either with saline or GH at early ages. Scale bar: 100 μm. Quantification of GFAP staining represents number of GFAP‐positive cells per field (error bars indicate SEM) in the region of the ARC (*n* = 4 to 6 mice/group), *P < 0.05 versus control.

We evaluated two indices of hypothalamic inflammation: GFAP (glial fibrillary acidic protein, a marker of astrocyte activation) and production of TNF‐α by microglia. Immunofluorescent staining for GFAP was less intense in the ARH of 18‐month‐old Ames male mice (Fig. 3) compared to littermate control mice (*P* < 0.05), consistent with other work associating long lifespan with lower hypothalamic inflammation (Zhang *et al*., 2013). Thus, Ames dwarf mice resemble CL mice in that both mice have lower levels of hypothalamic inflammation. Early‐life treatment of Ames dwarf mice with GH, however, restored the number of GFAP+ cells to the level seen in age‐matched control mice. Mice given injections of saline, rather than GH, showed no such effect (Fig. 3).

**Figure 4:**
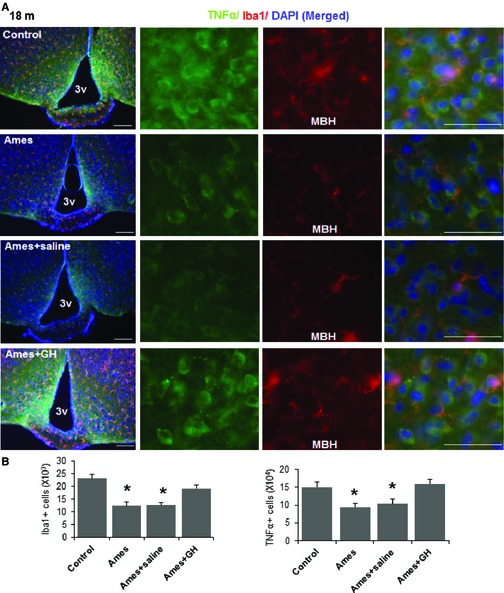
Hypothalamic inflammation in GH‐treated Ames mice. Brain sections of 18‐month‐old mice were analyzed for hypothalamic microglia and TNF‐α. (A) Representative images showing immunostaining in the MBH subregion of control and Ames dwarf mice, and in Ames mice that had been treated either with saline or GH. Scale bars: 100 μm (far left); 20 μm (right side panels).(B) Numbers of cells immunoreactive for Iba‐1, or TNF‐α in the hypothalamic mediobasal region (across the confocal microscopic field of serial sections) from indicated male mice (*n* = 4 to 6/group); error bars show SEM *P < 0.05 versus control.

To evaluate cytokine production by microglial cells, we stained for TNF‐α in the mediobasal hypothalamus (MBH) of 18‐month‐old mice and co‐stained for Iba‐1 as a marker for microglia. The number of immunoreactive TNF‐α‐positive cells was lower in Ames dwarf mice than in littermate controls, consistent with the GFAP data. Early‐life GH injections prevented this effect; such mice did not differ from control mice not bearing the Ames dwarf mutation (Fig. 4). Mice that had received saline instead of GH injections closely resembled uninjected Ames dwarf mice when evaluated at 18 months of age (Fig. 4B).

### Liver‐specific deletion of GHR does not modulate hypothalamic α‐MSH and AgRP fiber density

**Figure 5:**
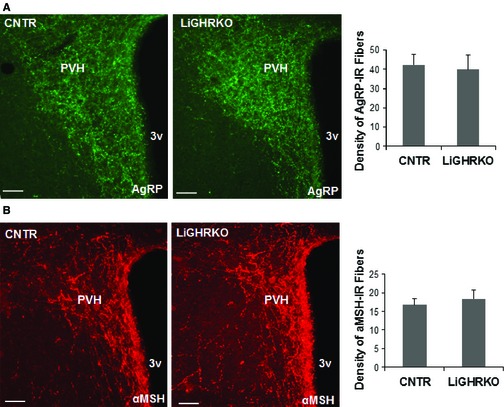
Images and quantification of agouti‐related peptide (AgRP) (A) and α‐melanocyte‐stimulating hormone (α‐MSH)‐immunoreactive fibers (B) innervating the paraventricular nucleus of the hypothalamus (PVH) at 6‐month‐old control and LiGHRKO male mice; (*n* = 4/group). 3V, third ventricle (Bregma: ‐0.82). Scale bar: 100 μm. Error bars reflect SEM.

Mice in which GHR is disrupted only in liver have 90% lower levels of serum IGF‐1 compared to controls, but do not show lifespan extension in either of two separate colonies (Dominick *et al*., 2015). GH action in other organs is, however, intact, and the mice have elevated serum GH as a result of disruption of IGF‐1‐dependent feedback circuits (List *et al*., 2014). We found that the density of AgRP and α‐MSH fibers in adult 6‐month‐old LiGHRKO male mice was comparable to that of control littermates (Fig. 5), suggesting that the changes in hypothalamic neuron projection density in GHR^−/−^ mice were not caused by lower plasma IGF‐1 levels. Similarly, hypothalamic GFAP and TNF‐α were comparable between 6‐month‐old LiGHRKO mice and control littermates (Fig. S1).

### Effect of GH deficiency on hypothalamic leptin signaling

**Figure 6:**
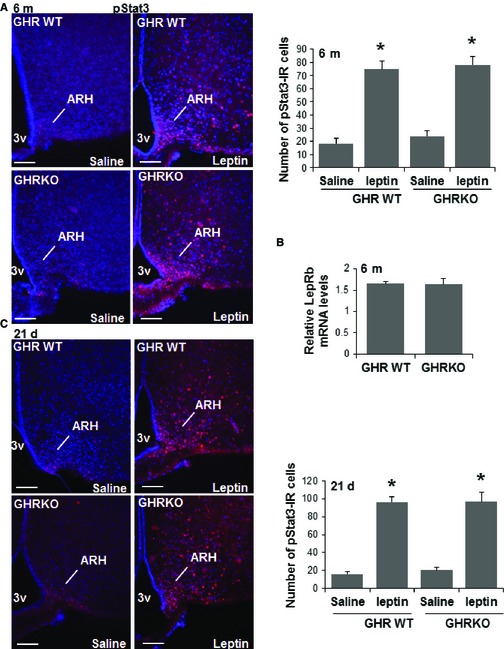
Hypothalamic leptin signaling in GHR ^−/−^ mice. Immunostaining for pStat3 1 h after intraperitoneal injection of vehicle or leptin (5 mg kg^−1^), with quantification of pStat3 immunoreactivity in (A) 6‐month‐old male mice and (C) 21‐day‐old male mice of the indicated groups (B) LepRb mRNA levels in 6‐month‐old male mice, 3V = third ventricle. Representative images from the hypothalamus are shown. Scale bar: 100 μm. (*n* = 4/each group), error bars reflect mean ± SEM. *P < 0.05 versus WT.

Crowded litter mice are also hypersensitive to leptin signals, as measured by leptin‐induced increase in phosphorylation of Stat3 (Sadagurski *et al*., 2015). GHR^−/−^ mice are obese, with elevated leptin levels, and leptin is known to be critical for formation of ARH projections (Baquedano *et al*., 2014). We therefore assessed whether alteration of projection pathways from the ARH in GHR^−/−^ mice was due to reduced ability of leptin to promote ARH neurite extension. The leptin‐stimulated accumulation of pStat3 in the brain reflects cell‐autonomous LepR‐b‐induced intracellular signaling, which is impaired in states associated with diminished leptin action (Leinninger & Myers, 2008). The LepR‐b/Stat3 pathway plays a role in early circuit formation and controls the architecture of POMC, but not AgRP, projections (Bouret *et al*., 2012). Peripheral injection of leptin in 6‐month‐old GHR^−/−^ mice robustly induced pStat3 immunoreactivity in the ARH after 1 h, similar to levels seen in the control mice (Fig. 6A). In addition, we detected a similar expression pattern of the LepR‐b mRNA in the GHR^−/−^ and control mice at this age (Fig. 6B). Furthermore, peripheral injection of leptin similarly induced pStat3 in GHR^−/−^ and control mice at 21 days of age (Fig. 6C). These results suggest that leptin‐induced intracellular signaling is not disturbed in GHR‐deficient mice and that the effects of GH on the architecture of ARH circuitry are not due to changes in leptin responses.

## Discussion

We demonstrate here that three strains of dwarf mice (Ames, Snell, and GHR^−/−^) in which low GH action lead to lifespan extension have diminished density of both AgRP and α‐MSH fibers in the hypothalamus and lower levels of hypothalamic inflammation. Strikingly, these effects are prevented by transient early‐life GH treatment in at least Ames dwarf mice that also blocks the lifespan effect. Neither effect is seen in mice with liver‐specific disruption of GHR, which do not show the longevity increase found in mice with global disruption of GHR. These data suggest that the hypothalamus may make a critical contribution to the lifespan effect in these mice. Because long‐lived CL mice show changes in hypothalamic inflammation that parallel those reported here for GH mutant mice, but opposite changes in hypothalamic neuronal projection (Sadagurski *et al*., 2015) and because inhibition of hypothalamic inflammation has been reported to increase mouse lifespan (Zhang *et al*., 2013), we suspect that the decline in inflammation may be the more important factor for lifespan extension.

We demonstrate a significant decrease in the density of both orexigenic and anorexigenic neurite projections in Snell, Ames, and GHR^−/−^ dwarf mice, compared with normal mice, at ages ranging from 5–6 months to 18 months. Our data are consistent with previous reports that hypothalamic mRNA levels of NPY, AgRP, and POMC are decreased in Ames and Snell mice, compared to controls (Peng *et al*., 2001; Hurley *et al*., 2003). Similarly, hypothalamic mRNA levels of AgRP, POMC, and NPY are reduced in GHR^−/−^ mice, with reduced inflammation in response to high‐fat diet (HFD) (Baquedano *et al*., 2014). The absence of GH action could plausibly underlie the decline in NPY and AgRP levels, because these neurons express GHR and require GH for growth (Chan *et al*., 1996; Bohlooly *et al*., 2005). Leptin and insulin are important anorexigenic hormones that signal to the hypothalamus, and both NPY/AgRP and POMC/CART neurons express receptors for these two hormones (Bouret & Simerly, 2007). We see no changes in hypothalamic responses to leptin, as measured by Stat3 phosphorylation, or in expression of LepR‐b in GHR^−/−^ mice (Fig. 6). These data indicate that the GHR, which is expressed and competent during the time of the postnatal leptin surge, mediates the developmental actions of GH on ARH projections. Notably, both AgRP‐ and POMC‐containing projections appear affected by GH and GHR deficiency.

Changes in hypothalamic architecture that occur in the immediate postnatal period appear to be malleable, and are particularly susceptible to changes in hormonal signals (Bouret & Simerly, 2006). Consistent with this idea, Ames dwarf mice subjected to daily GH injections starting at the age of 2 weeks for 6 weeks showed a remarkable reduction in longevity, so that their lifespans no longer differed from that of genetically normal littermates (Panici *et al*., 2010). In contrast, GH treatment of Snell dwarf mice, when started at 4 weeks, did not influence lifespan (Vergara *et al*., 2004). GH injections started at 4 weeks of age also failed to reduce longevity in Ames dwarf mice (Bartke, unpublished). Although these protocols differed in important ways, including the intensity of the GH injection schedule, the disparities suggest that sensitivity of lifespan to GH signals may change between 2 weeks and 4 weeks of age in mice. Our new results show that Ames dwarf mice that had been subjected to a series of GH injections between 2 and 8 weeks of age did not show the decline in ARH neuronal projections in PVH seen in uninjected or saline‐injected Ames dwarf mice. Furthermore, hypothalamic inflammatory changes observed in Ames dwarf mice were reversed. Thus, a few weeks of exposure to systemic GH, early in postnatal life, leads to permanent changes in the hypothalamic circuitry and inflammatory responses.

Our findings suggest that the changes in hypothalamic branching patterns and midlife inflammatory tone seen in Ames and Snell dwarf mice are mediated principally by early‐life exposure to systemic GH, rather than to defects in prolactin or thyroid hormones (Masternak & Bartke, 2012). Food consumption, O2 utilization, when adjusted for body mass, is higher in dwarf and GHR^**−/−**^ mice, and these mice also have higher adiposity (Meyer *et al*., 2004; Bonkowski *et al*., 2006). It is possible that their elevations in adiponectin and leptin, or reduced body temperature, contribute to their extended longevity (Bartke *et al*., 2013) and may themselves be influenced by alterations in hypothalamic status.

The absence of GH action, either globally or in mice with liver‐specific GH knockout, impairs liver IGF‐1 synthesis, allowing discrimination between direct effects of GH and those mediated by endocrine IGF‐1. Our data show that changes in hypothalamic neurite density seen in mice with global GHR deletion are not replicated in mice with liver‐specific disruption of GHR, suggesting that the hypothalamic changes are not brought about by lower plasma IGF‐1 levels. Disruption of IGF‐1 gene expression during early life causes growth retardation and defects in the development of metabolic organs that can alter energy homeostasis throughout adult life (Woods *et al*., 1996). GHR^**−/−**^ mice have increased expression of hypothalamic IGF‐1 and IGF1R (Nyberg, 2000; Bartke, 2011), despite lower levels of plasma IGF‐1. Enhanced hypothalamic IGF‐1 expression in the mice with global disruption of GHR raises the possibility that compensatory increases in the local production and paracrine/autocrine actions of IGF‐1 could account for some characteristics of GH‐deficient mouse strains, because both GH and IGF‐1 exert neuroprotective effects (Sonntag *et al*., 2005). Liver‐specific GHR knockout mice (LiGHRKO) have a 90% decrease in circulating IGF‐1 levels and a 300% increase in circulating GH (List *et al*., 2014), although no data are available on the levels of IGF‐1 and IGF1R in the hypothalamus of LiGHRKO mice. Unaltered ARH circuitry in the LiGHRKO mice demonstrates a specific role of GH action in hypothalamic development.

Hypothalamic inflammation influences the set points of multiple homeostatic loops. A recent publication (Zhang *et al*., 2013) has shown that modulation of inflammatory circuits in the hypothalamus, involving NF‐kB/IKKb signals under the control of GnRH, can extend lifespan and delay multiple aspects of aging in mice. Long‐lived GH‐deficient and GH‐resistant mice have reduced levels of IL‐6 and TNF‐α, mediators of inflammation (Bartke, unpublished). Further, hypothalamic IL‐1β expression in Ames dwarf mice is significantly reduced in comparison with normal control animals (Bartke, unpublished). It was previously reported that GHR^**−/−**^ mice have higher serum levels of TNF‐α and IL‐1 as compared to control mice independently of diet. The increase in serum inflammatory markers in GHR^**−/−**^ mice was coincident with an inflammatory pattern in the hypothalamus. However, their mRNA levels were decreased in the hypothalamus suggesting that there may be increased uptake of these cytokines from the circulation (Baquedano *et al*., 2014). We have now found that hypothalamic inflammation is reduced in middle‐aged mice by the Ames dwarf mutation and that transient early‐life exposure to GH was able to reverse this phenotype.

End‐of‐life necropsy data have been published for Ames, Snell, and GHR^**−/−**^ mice (Ikeno *et al*., 2003, 2009; Vergara *et al*., 2004), showing that the extension of lifespan is not accompanied by a major shift in the relevant proportions of specific lethal illnesses. Most of the mutant mice, such as mice in the control groups, die of some form of neoplasia, rather than of metabolic diseases such as diabetes or atherosclerosis. Connections between hypothalamic inflammatory set points and lifelong cancer risk are obscure, but clearly deserve further investigation.

Nutritional excess is a key activator of metabolic inflammation in the hypothalamus, suggesting that interventions that can counteract hypothalamic neuroinflammation may protect against metabolic disorders and diabetes. The discovery that inhibition of hypothalamic inflammation can delay aging (Zhang *et al*., 2013) suggests that drugs that extend lifespan might be effective in inhibiting centrally regulated inflammatory processes. It will be important to determine the effects of drugs that extend mouse longevity, such as aspirin (Strong *et al*., 2008), rapamycin (Miller *et al*., 2011), and nordihydroguaiaretic acid, acarbose, and 17‐α‐estradiol (Harrison *et al*., 2014) on hypothalamic inflammatory processes elevated with age.

## Methods

### Animals

Procedures involved in this study were approved by the University of Michigan Committee on the Use and Care of Animals (UCUCA). GHRKO mice on a C57BL/6J background and homozygous WT littermates were used (Chandrashekar *et al*., 1999). Genotype was determined by PCR analysis of genomic DNA obtained from a tail sample collected at weaning. Ames dwarfs (Prop1^df^) and homozygous mice (df/df) were produced on a genetically heterogeneous background by mating heterozygous females and homozygous mutant males at Southern Illinois University, and sent to the University of Michigan. In this colony, the Prop1^df^ mutation is maintained on a heterogeneous genetic background. Liver tissue‐specific GHR^−/−^ (LiGHRKO) mice were generated as described previously (List *et al*., 2014). Animals were maintained under temperature‐ and light‐controlled conditions (20–23 °C, 12‐h light–dark cycle).

### Growth hormone treatment

Groups of 15–17 Ames dwarf males were subjected to treatment with porcine GH (pGH) via subcutaneous (s.c.) injection [4 μg g^−1^ body weight (bw) per day], given 2× per day starting at the age of 2 weeks and continuing for 6 weeks. Twice‐daily saline‐treated dwarfs (df/df) of the same age were used as controls. After 6 weeks of treatment, animals were maintained normally and euthanized at 18 months of age. These mice were not exposed to any other manipulations.

### Leptin treatment

For peripheral leptin treatment, mice were injected i.p. with either 5 mg kg^−1^ recombinant mouse leptin (provided by Dr. A Parlow, National Hormone and Pituitary Program, Torrance, CA, USA) or vehicle as previously described (Sadagurski *et al*., 2012). Mice were sacrificed 1 h after an i.p. injection of leptin or vehicle performed in overnight‐fasted animals.

### RNA extraction and qPCR

Hypothalami were carefully dissected using Brain Matrices (Braintree Scientific, Braintree, MA, USA). Isolated mRNA from this tissue was analyzed using quantitative real‐time PCR. RNA was isolated using the Qiagen RNeasy Kit (Qiagen, Valencia, CA, USA), which was combined with the RNase‐Free DNase Set (Qiagen). RNA was reverse‐transcribed with the High Capacity cDNA RT Kit and amplified using TaqMan^®^ Universal PCR‐Master Mix, NO AmpErase UNG with TaqMan^®^ Assay‐on‐demand kits (Applied Biosystems, Foster City, CA, USA). Relative expression of target mRNAs was adjusted for total RNA content by beta‐actin RNA quantitative PCR. Quantitative PCR was performed on an ABI‐PRISM 7900 HT Sequence Detection system (Applied Biosystems). Each reaction was carried out in triplicates as previously described (Sadagurski *et al*., 2010). Primers used including the following: *AgRP* F: AGGGCA TCAGAAGGCCTGACCA, *AgRP* R: CTTGAAGAAGCGGCAGTAGCAC, *POMC* F: AAGAGCAGTGACTAAGAGAGGCCA, *POMC* R: ACATCTATGGAGGTCTGAAGCAGG, *NPY* F: TAGGTAACAAACGAATGG GG, *NPY* R: AGG ATGAGATGAGATGTGGG, LepR‐b R: GGAGACAGAGGCCCAGACATT, LepR‐b F: AAACTTCCCTCCAGTTCCAAAAG‐3.

### Perfusion and immunolabeling

Mice were anesthetized (IP) pentobarbital and transcardially perfused with phosphate‐buffered saline (PBS) (pH 7.5) followed by 4% paraformaldehyde (PFA). Brains were postfixed, dehydrated, and then sectioned coronally (30 μm) using a sliding microtome, followed by immunohistochemical or immunofluorescent analysis as previously described (Patterson *et al*., 2010). For immunohistochemistry, free‐floating brain sections were pretreated by sequential incubations in 0.3% H_2_O_2_/1% NaOH, 0.3% glycine, and 0.03% SDS, followed by blocking in normal donkey serum (NDS). Sections were incubated with goat anti‐AgRP (1:1000; Phoenix Pharmaceuticals, Belmont, CA, USA), sheep anti‐α‐MSH (1:1000; Millipore, Temecula, CA, USA), rabbit anti‐GFAP (1:1000; Millipore), mouse anti‐TNF‐α (1:500; Abcam, Cambridge, MA, USA), or rabbit anti‐Iba‐1 (1:1000; Wako, Richmond, VA, USA) as primary antibodies, followed by Alexa Fluor‐conjugated secondary antibodies (Invitrogen, Carlsbad, CA, USA) as previously published (Munzberg *et al*., 2004; Bouret *et al*., 2008; Sadagurski *et al*., 2012; Zhang *et al*., 2013; Vogt *et al*., 2014). For pStat3 immunostaining, sections were pretreated for 20 min in 0.5% NaOH and 0.5% H_2_O_2_ in potassium PBS, followed by immersion in 0.3% glycine for 10 min. Sections were then placed in 0.03% SDS for 10 min and placed in 4% normal serum plus 0.4% Triton X‐100 plus 1% BSA for 20 min before incubation for 48 h with a rabbit anti‐pStat3 antibody (1:1000; Cell Signaling Technology, Danvers, MA, USA). Sections were mounted onto Superfrost Plus slides (Fisher Scientific, Hudson, NH, USA) and cover slips added with ProLong Antifade mounting medium (Invitrogen). Microscopic images were obtained using an Olympus FluoView 500 Laser Scanning Confocal Microscope (Olympus, Center Valley, PA, USA) equipped with a 20× objective.

### Quantification

The density of AgRP and α‐MSH innervation of the PVH (bregma:‐0.82), DMH (bregma:‐1.58), and ARH (bregma:‐1.8) was determined by quantitative confocal microscopy using previously published methods (Patterson *et al*., 2010). For each animal, two sections through the PVH were acquired. Image analysis was performed using Image J analysis software (version 1.39t; National Institutes of Health, Bethesda, MD, USA). Each image plane was binarized to isolate the labeled fibers from the background as well as to compensate for differences in fluorescence intensity and was then skeletonized so that each fiber segment was 1 pixel thick. The integrated intensity was then calculated for each image, which reflects the total number of pixels in the skeletonized image and was proportional to the total length of labeled fibers in the image. This procedure was carried out on each image plane in the stack, and the values for all image planes in a stack were summed. The resulting value is an accurate index of fiber density in the volume sampled (Patterson *et al*., 2010).

For quantification of immunoreactive neurons, images of matched brain areas were taken from at least three sections containing the ARH of the hypothalamus for each brain between bregma −1.3 mm and −2.4 mm (according to the Franklin mouse brain atlas). Serial brain sections across the hypothalamus were made at 20 μm thickness, and every five sections were represented by one section with staining and cell counting. All sections were arranged from rostral to caudal to examine the distribution of labeled neurons. The images were quantified with Imaris (versions 6.4 and 7.0; Bitplane). The number of positive neurons was presented as means ± SEM.

### Statistical analysis

Data sets with more than two groups were analyzed using one‐way analysis of variance (anova) followed by Tukey's post hoc test. For GH injections experiments, the statistical comparison of the untreated mutant mice was with untreated genetic controls, and the comparison of the GH‐treated mutant mice was with the saline‐injected mutant controls. Two‐tailed Student's *t*‐tests were used for comparisons involving only two groups. All data were presented as mean ± SEM. *P* < 0.05 was considered significant.

## Funding

This project was supported by NIH grant R01‐AG019899 (to AB), by a Senior Scholar Award from the Ellison Medical Foundation (to RAM) and by the UM Nathan Shock Center, P30‐AG013283 (to RAM). MS was supported by a pilot grant from Shock Center AG‐13283, the Pepper Center AG‐024824, and a Feasibility Grant from the Michigan Diabetes Research Center (P30DK020572).

## Conflict of interest

No conflict of interests, financial or otherwise, are declared by the authors.

## Supporting information


**Fig. S1** Hypothalamic inflammation in LiGHRKO mice.
