# Supplementary figures and images for: Growth hormone modulates hypothalamic inflammation in long‐lived pituitary dwarf mice
Source: Aging Cell. 2015 Aug 12;14(6):1045–54. doi: 10.1111/acel.12382 (PMC4693470; doi:10.1111/acel.12382)

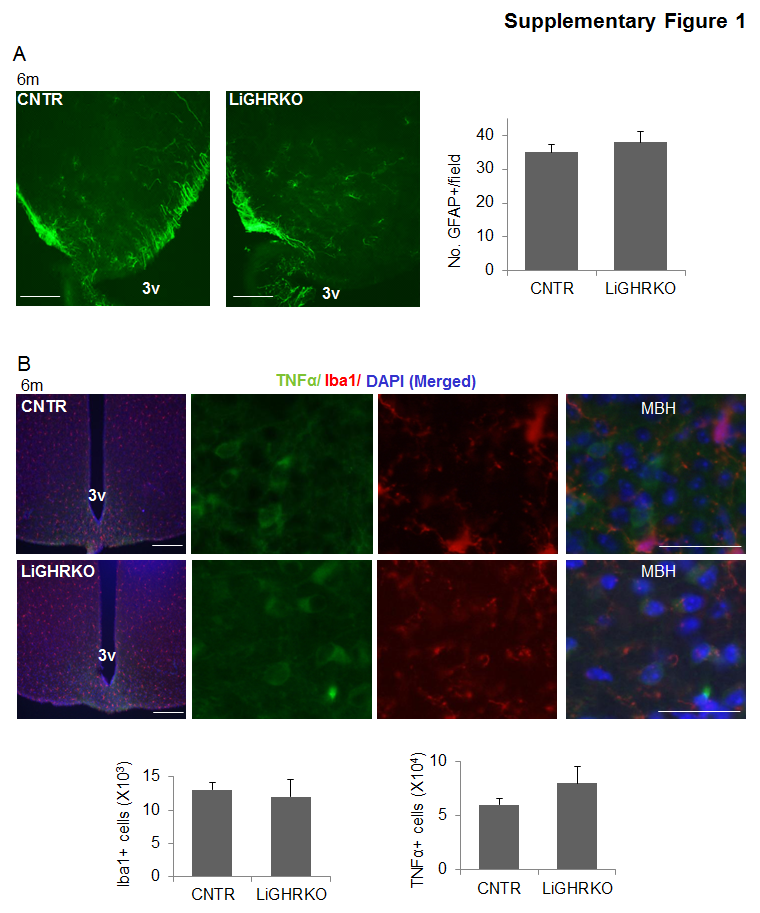

Supplement: Supplementary file 1 — Fig. S1 Hypothalamic inflammation in LiGHRKO mice. [file ACEL-14-1045-s001.tif]
